# No Significant Radiological Signs of Adult Spinal Deformity Progression after a Mean of 11 Years of Follow-Up Following Harrington Rod Instrumentation Removal and Watchful Waiting

**DOI:** 10.3390/healthcare11081149

**Published:** 2023-04-17

**Authors:** Peter Brumat, Janez Mohar, Dejan Čeleš, Danijel Erdani, Nikša Hero, Matevž Topolovec

**Affiliations:** 1 Valdoltra Orthopaedic Hospital, 6280 Ankaran, Slovenia; 2 Faculty of Medicine, University of Ljubljana, 1000 Ljubljana, Slovenia; 3 Faculty of Medicine, University of Maribor, 2000 Maribor, Slovenia

**Keywords:** adult spinal deformity, Harrington rod instrumentation, spinal instrumentation removal, watchful waiting, long-term outcome, radiographic outcome

## Abstract

The study aimed to assess long-term radiological outcomes in patients from our institution who were primarily treated for adolescent idiopathic scoliosis with surgical correction using Harrington rod (HR) instrumentation, and afterward with watchful waiting of residual spinal deformity after HR removal, whereby no patient consented to spinal deformity correction. A single-institution case series of 12 patients was retrospectively evaluated. Preoperative and most recent post-instrumentation removal radiographic measurements were compared, along with baseline characteristics. The average age of patients (all females) at the time of HR instrumentation removal was 38 ± 10 years (median 40, range 19–54). The mean follow-up from the HR instrumentation implantation to the HR instrumentation removal was 21 ± 10 years (median 25, range 2–37), with a further mean of 11 ± 10 years (median 7, range 2–36) of follow-up following HR instrumentation removal and watchful waiting. No significant change in radiological parameters was observed: LL (*p* = 0.504), TK (*p* = 0.164), PT (*p* = 0.165), SS (*p* = 0.129), PI (*p* = 0.174), PI–LL (*p* = 0.291), SVA (*p* = 0.233), C7-CSVL (*p* = 0.387), SSA (*p* = 0.894), TPA (*p* = 0.121), and coronal Cobb angle (proximal (*p* = 0.538), main thoracic (*p* = 0.136), and lumbar (*p* = 0.413)). No significant change in coronal or sagittal parameters was observed in this single-institution long-term radiological outcome study of adults following HR instrumentation removal and watchful waiting of residual spinal deformity.

## 1. Introduction

Instrumentation with Harrington rod (HR) used to be the standard of care for scoliosis treatment in children in the past [1]. Such a method of spinal instrumentation is frequently associated with residual spine deformity in adults, whereby iatrogenic sagittal malalignment presents one of the most recognized causes of adult spinal deformity (ASD) [1,2,3]. The disorder is of growing interest in health care, given the prevalence of ASD in the expanding portion of the global population aged older than 65 years [2]. Moreover, with a substantial impact on quality of life, the prevalence of ASD is reported to be as high as 68% in patients older than 60 years [4].

ASD can be managed either with nonsurgical methods or surgical treatment. The nonoperative treatment is regarded as the first-line response, whereas surgical intervention has recently become the mainstay of treatment, since it may yield more favourable outcomes than nonsurgical methods for improving the function of patients with ASD and achieving good pain improvement and deformity correction [2,3,5]. However, surgical correction for HR instrumentation sequelae presents a challenge in ASD surgery due to complex conditions involving residual structural alterations and technically demanding procedures frequently requiring osteotomies, with the high surgical risk involved [1,6,7]. The surgical treatment of ASD results in a substantial financial burden on the healthcare system and resource utilization as well [4,8]. Despite the fact that ASD is a complex entity that has had emerging significance for spine surgeons in the last decade, global disparities exist in both the assessment and treatment of adults with spinal deformity across countries of varying incomes, which represents an area requiring further investigation [2,6]. Regardless of the chosen treatment modality, the management of ASD is aimed at maintaining a well-aligned spine in all planes.

The purpose of this study was to assess long-term radiological outcomes in patients from our institution who were treated primarily for adolescent idiopathic scoliosis with surgical correction using HR instrumentation, and afterward with conservative management (watchful waiting) of residual spinal deformity after HR removal, whereby no patient consented to adult spinal deformity correction.

## 2. Materials and Methods

**Figure 1:**
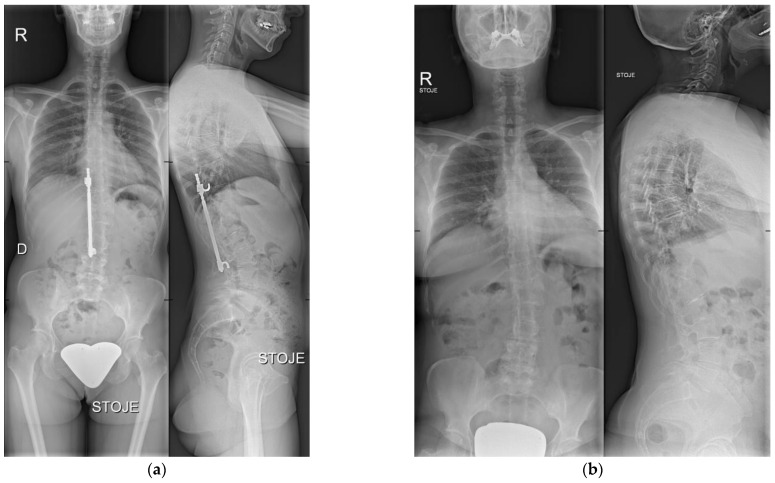
(**a**) X-ray taken before HR instrumentation removal. Anteroposterior (**left**) and lateral (**right**) view. (**b**) Most recent X-ray after HR instrumentation removal. Anteroposterior (**left**) and lateral (**right**) view.

We performed a retrospective, institutional review board-approved (No. 2/2022) analysis of a single-institution case series of 12 consecutive patients with adolescent idiopathic scoliosis, treated primarily with surgical correction using HR instrumentation (Figure 1a) between 1982 and 1997, and afterward with watchful waiting of residual spinal deformity after HR instrumentation removal (Figure 1b), whereby no patient consented to surgical residual deformity correction. Written informed consent from the patients was obtained.

The upper instrumented vertebra (UIV) was in five cases Th5, in two cases Th4, and in one case Th2, Th3, Th6, Th7, and Th10. The lower instrumented vertebra (LIV) was in one case Th12 and L1, in two cases L3, and in eight cases L4. The indication for instrumentation removal was metal hypersensitivity in one case, instrumentation failure in one case, progressive back pain and/or radiculopathy in six cases, and an indication for a diagnostic head magnetic resonance imaging (MRI) in four cases. Other patients who underwent concomitant instrumented fixation without deformity correction at the time of HR removal (7 patients) were not included in the analysis.

Demographic data collected included age, sex, body mass index (BMI), and the American Society of Anesthesiologists (ASA) physical status score. Radiological measurements included lumbar lordosis (LL), thoracic kyphosis (TK), pelvic tilt (PT), sacral slope (SS), pelvic incidence (PI), pelvic incidence–lumbar lordosis (PI–LL), sagittal vertical axis (SVA), C7-central sacral vertical line (C7-CSVL), spinosacral angle (SSA), T1 pelvic angle (TPA), and coronal Cobb angle (proximal, main thoracic, and lumbar, where applicable) before HR instrumentation removal and most recently after HR instrumentation removal and watchful waiting.

### Data Analysis

The Shapiro–Wilk test was used to assess for normal distribution. Pre- and post-instrumentation removal radiographic measurements were performed in Agfa Impax version 6 (Agfa-Gevaert, Mortsel, Belgium) and Surgimap version 2.3.2.1 (Surgimap, Methuen, MA, USA) software, and compared using two-tailed paired Student’s *t*-test and Wilcoxon signed-rank test. No multiple comparison corrections were applied to statistical significance testing. Statistical analyses were performed in R software 4.1.3 (R Foundation for Statistical Computing, Vienna, Austria). *p* < 0.05 was considered significant.

## 3. Results

The average age of patients (all females) at the time of HR instrumentation removal was 38 ± 10 years (median 40, range 19–54), the average BMI was 25 ± 6 (median 24, range 18–41), and the average ASA score was 2 ± 1 (median 2, range 1–3). No postoperative complications were observed. The mean follow-up from the HR instrumentation implantation to the HR instrumentation removal was 21 ± 10 years (median 25, range 2–37), with a further mean of 11 ± 10 years of follow-up (median 7, range 2–36) following HR instrumentation removal and watchful waiting, and a minimum of 2 years of follow-up after HR instrumentation removal for each patient (Table 1). The averages with standard deviations of the pre- and post-HR instrumentation removal radiographic measurements are reported in Table 2. No significant change in radiological parameters was observed, as reported in Table 2.

## 4. Discussion

This study aimed to assess long-term radiological outcomes in patients from our institution who were treated primarily for adolescent idiopathic scoliosis with surgical correction using HR instrumentation (Figure 1a), and afterward with watchful waiting of residual spinal deformity after HR instrumentation removal, whereby no patient consented to adult spinal deformity correction (Figure 1b). The results showed no significant radiological signs of ASD progression after an average follow-up of 11 years after the HR instrumentation removal.

In recent years, the importance of a well-aligned spine has been increasingly recognized, regardless of the spine pathology [9]. Measurement of certain spinal coronal and sagittal radiological parameters has been recognized as crucial for the analysis of spinal deformities (including ASD) and planning of the treatment [3]. Based on the tight relationship between sagittal plane deterioration and quality-of-life measures, Schwab et al. [10] incorporated sagittal parameters into an adult spine deformity classification system and determined cutoff values for the most clinically relevant parameters in the sagittal profile (SVA, PI–LL, and PT) based upon multicenter data [11]. The global sagittal alignment of the spine, the degree of LL and the (mis)match between the latter and the PI may directly affect the health-related outcomes associated with quality of life, such as pain and disability [1,3,9]. HR instrumentation has been connected to subsequent sagittal malalignment of the spine, particularly due to the loss of LL, and further development of ASD, despite its positive impacts on the correction of the coronal-plane alignment [1,3]. In the case of HR instrumentation, the obliteration of LL with subsequent increase of PI–LL mismatch and SVA leads to pelvic retroversion (greater PT) and a more horizontally placed sacrum (lower SS) to compensate for the mismatch [1,3,9]. The second mechanism of spinal compensation consists of hyperextension of the spinal segments outside of the instrumented area to remain upright, which induces the flattening of the TK and hyperextension of the neck, ensuing the so-called “flatback syndrome” [1,9].

In our study, we analyzed the long-term effect of the potential progression of radiological spinal parameters in the interval from the HR instrumentation removal to the last follow-up after HR instrumentation removal and watchful waiting. We observed a progression of SVA from the mean 18 mm to the mean 33 mm, loss of LL from the mean 39° to the mean 35°, PI–LL progress from the mean 11 to the mean 16, PT progress from the mean 18° to the mean 23°, which may be within (SVA, PI–LL, PT) the recommended ideal target values for sagittal alignment [10,11,12]. It is suggested that the ideal PI–LL may be between 10° and 20° in adult degenerative scoliosis patients after long posterior instrumentation and fusion [12]. Although not statistically significant, we noticed a reduction of the average TK and SS values from 27° to 23°, and 33° to 27°, respectively, and in line with the literature [1,3,9]. UIV was in five cases Th5 (42%), and LIV was in the majority of our cases (67%) L4, which may additionally explain the sagittal malalignment. Some authors further describe a specific surgical technique to avoid subsequent sagittal malalignment associated with the HR implantation [13], but this technique was not implemented at our institution. In line with the literature [1,3], we also observed a slight progression of the average coronal Cobb angle and C7-CSVL value (proximal Cobb angle 17° to 19°, main thoracic Cobb angle 32° to 35°, lumbar Cobb angle 30° to 32°, and C7-CSVL 17 mm to 21 mm), whereas no significant changes of coronal spinal parameters (coronal Cobb angle and C7-CSVL) were seen. A significant coronal imbalance of greater than 4 cm may be associated with deterioration in pain and function scores for unoperated patients [14]. Because the positioning of the C7 in relation to the sacrum may impact the SVA, the angular and/or ratio parameters to characterize the positioning of C7 in relation to the sacrum are recommended [15]. Additionally, the T1 pelvic angle (TPA) was introduced as a measure of sagittal alignment independent of postural compensatory mechanisms [16]. In our study, the average SSA and TPA values progressed from 115° to 120° and from 17° to 21°, whereby detected TPA was borderline for the TPA threshold for poor alignment [16,17,18]. Again, this radiological progression observed in our study was also not significant. However, this may suggest that, when dealing with a fused spine or rigid spinal deformity, the measurement of only the linear radiological parameters instead of also/or the angular ones may suffice for the evaluation of the global spinal profile.

The surgical treatment for ASD has become increasingly common, despite concerning surrounding complications, revision rates, and cost-effectiveness [8]. Major benefits of surgical treatment have been shown for adults with painful and disabling spinal deformity, although the optimal operative treatment may be highly dependent on the patient’s symptomatology and is surgeon-dependent [4]. During surgical planning, a thorough assessment of the patient-specific radiological parameters of the deformity, both in the sagittal and coronal plane, is essential, especially global (sagittal) alignment parameters, PI–LL mismatch and PT; the latter two being the quantifiers of the loss of LL, which is generally seen as the main “deformity driver” [3]. Achieving global coronal and sagittal alignment is an independent predictor of both satisfaction and disability at 2 years post-op [19]. Nevertheless, realignment procedures should respect deformity and age-adjusted alignment targets [11,20].

The level of evidence regarding the effectiveness of conservative treatment in patients with ASD is low; however, patients with mild disability and pain are often encouraged to try the conservative treatment modalities before the surgical ones [3]. This may be indicated when dealing with patients who have mean regional (PI–LL) and segmental alignment objectives in the context of global alignment (TPA/SVA) and PT, as was observed in our study. The choice for conservative treatment may be also dependent upon the healthcare system of a specific country, but in general, the patient’s decision for either conservative or surgical treatment of an ASD is influenced by the degree of pain and disability, especially in older patients [2,3]. Overall, the planning of the (surgical) treatment of the patient with ASD must consider multiple variables, such as the patient’s age, comorbidities, expectations, and possible complications to improve the patient’s quality of life post-treatment [1,3,9]. Despite the well-established options for surgical treatment of ASD, some patients may also willingly opt for conservative management, hypothetically owing to the noteworthy complications possibly incurred by the surgical treatment. Thus, the results of our study suggest that adults with no significant change in coronal or sagittal parameters with long-term follow-up following Harrington rod instrumentation removal and watchful waiting may be amenable to conservative management of residual spinal deformity if surgical correction of spinal deformity is not consented to.

This study has some limitations. One limitation is that we only assessed the long-term radiological outcome, which revealed no significant deformity progression after a mean of 11 years of follow-up. However, patient-reported outcome measures would have gauged also the clinical outcome of the same follow-up and allowed for further statistical comparison, whether there is any impact on quality of life. Another limitation is a small case series, but our study reports on a long-term radiographic outcome of ASD treatment in a specific population from a single-institution following HR instrumentation removal. Multicentric studies are needed for clarification.

## 5. Conclusions

No significant change in coronal or sagittal parameters was observed in this single-institution long-term radiological outcome study of adults following Harrington rod instrumentation removal and watchful waiting of residual spinal deformity. Further studies are needed for clarification.

## Figures and Tables

**Table 1:** Baseline characteristics.

Patient #	Sex	Age at HR Instrumentation Implantation (Years)	Instrumentation (UIV–LIV)	Age at HR Instrumentation Removal (Years)	Follow-Up after HR Instrumentation Implantation to HR Instrumentation Removal (Years)	Follow-Up after HR Instrumentation Removal and Watchful Waiting (Years)
#1	Female	17	Th5-L4	19	2	36
#2	Female	24	Th5-L4	31	7	21
#3	Female	35	Th4-L4	54	19	21
#4	Female	11	Th3-Th12	28	17	15
#5	Female	11	Th2-L1	26	15	10
#6	Female	8	Th10-L3	33	25	8
#7	Female	13	Th7-L4	38	25	6
#8	Female	17	Th6-L4	41	24	5
#9	Female	14	Th4-L3	41	27	4
#10	Female	13	Th5-L4	50	37	3
#11	Female	15	Th5-L4	46	31	2
#12	Female	17	Th5-L4	43	26	2
Mean ± SD		16 ± 7		38 ± 10	21 ± 10	11 ± 10
Median (range)		15 (8–35)		40 (19–54)	25 (2–37)	7 (2–36)

Harrington rod (HR). Upper instrumented vertebra (UIV). Lower instrumented vertebra (LIV). Standard deviation (SD).

**Table 2:** Comparison of pre- and most recently post-HR instrumentation removal radiographic measurements.

	N (100%)	Average ± SD	*p*
LL (pre)	12 (100%)	39° ± 22°	0.504
LL (post)	12 (100%)	35° ± 27°	
TK (pre)	12 (100%)	27° ± 12°	0.164
TK (post)	12 (100%)	23° ± 11°	
PT (pre)	12 (100%)	18° ± 6°	0.165
PT (pre)	12 (100%)	23° ± 14°	
SS (pre)	12 (100%)	33° ± 12°	0.129
SS (post)	12 (100%)	27° ± 17°	
PI (pre)	12 (100%)	51° ± 14°	0.174
PI (post)	12 (100%)	50° ± 16°	
PI–LL (pre)	12 (100%)	11° ± 14°	0.291
PI–LL (post)	12 (100%)	16° ± 21°	
SVA (pre)	12 (100%)	18 ± 54 mm	0.233
SVA (post)	12 (100%)	33 ± 31 mm	
C7-CSVL (pre)	12 (100%)	17 ± 14 mm	0.387
C7-CSVL (post)	12 (100%)	21 ± 12 mm	
SSA (pre)	12 (100%)	115° ± 35°	0.894
SSA (post)	12 (100%)	120° ± 20°	
TPA (pre)	12 (100%)	17° ± 9°	0.121
TPA (post)	12 (100%)	21° ± 13°	
Coronal Cobb angle, proximal (pre)	7 (58%)	17° ± 7°	0.538
Coronal Cobb angle, proximal (post)	7 (58%)	19° ± 5°	
Coronal Cobb angle, main thoracic (pre)	11 (92%)	32° ± 11°	0.136
Coronal Cobb angle, main thoracic (post)	11 (92%)	35° ± 13°	
Coronal Cobb angle, lumbar (pre)	11 (92%)	30° ± 13°	0.413
Coronal Cobb angle, lumbar (post)	11 (92%)	32° ± 17°	

Lumbar lordosis (LL), thoracic kyphosis (TK), pelvic tilt (PT), sacral slope (SS), pelvic incidence (PI), pelvic incidence–lumbar lordosis (PI–LL), sagittal vertical axis (SVA), C7-central sacral vertical line (C7-CSVL), spinosacral angle (SSA), T1 pelvic angle (TPA), and coronal Cobb angle (proximal, main thoracic, and lumbar, where applicable). Before HR instrumentation removal (pre) and most recently after HR instrumentation removal and watchful waiting (post). Standard deviation (SD). Level of significance (*p*).

## Data Availability

Data are available upon reasonable request to the corresponding author.

## References

[1] Louie P.K., Iyer S., Khanna K., Harada G.K., Khalid A., Gupta M., Burton D., Shaffrey C., Lafage R., Lafage V. (2022). Revision Strategies for Harrington Rod Instrumentation: Radiographic Outcomes and Complications. Glob. Spine J..

[2] Diebo B.G., Shah N.V., Boachie-Adjei O., Zhu F., Rothenfluh D.A., Paulino C.B., Schwab F.J., Lafage V. (2019). Adult Spinal Deformity. Lancet.

[3] Kim H.J., Yang J.H., Chang D.-G., Suk S.-I., Suh S.W., Song K.-S., Park J.-B., Cho W. (2020). Adult Spinal Deformity: Current Concepts and Decision-Making Strategies for Management. Asian Spine J..

[4] Alvarado A.M., Schatmeyer B.A., Arnold P.M. (2021). Cost-Effectiveness of Adult Spinal Deformity Surgery. Glob. Spine J..

[5] Jia Y., Peng Z., Qin Y., Wang G. (2022). Surgical versus Nonsurgical Treatment for Adult Spinal Deformity: A Systematic Review and Meta-Analysis. World Neurosurg..

[6] Adler D., Almansour H., Akbar M. (2018). Was ist eigentlich eine adulte spinale Deformität?: Entwicklung, Klassifikation und Indikation zur operativen Therapie. Orthopäde.

[7] Kose K.C., Bozduman O., Yenigul A.E., Igrek S. (2017). Spinal Osteotomies: Indications, Limits and Pitfalls. EFORT Open Rev..

[8] Glassman S.D., Carreon L.Y., Shaffrey C.I., Kelly M.P., Crawford C.H., Yanik E.L., Lurie J.D., Bess R.S., Baldus C.R., Bridwell K.H. (2020). Cost Effectiveness of Adult Lumbar Scoliosis Surgery: An As-Treated Analysis from the Adult Symptomatic Scoliosis Surgery Trial with Five Year Follow-Up. Spine Deform..

[9] Cirillo Totera J.I., Fleiderman Valenzuela J.G., Garrido Arancibia J.A., Pantoja Contreras S.T., Beaulieu Lalanne L., Alvarez-Lemos F.L. (2021). Sagittal Balance: From Theory to Clinical Practice. EFORT Open Rev..

[10] Schwab F., Ungar B., Blondel B., Buchowski J., Coe J., Deinlein D., DeWald C., Mehdian H., Shaffrey C., Tribus C. (2012). Scoliosis Research Society—Schwab Adult Spinal Deformity Classification: A Validation Study. Spine.

[11] Diebo B.G., Varghese J.J., Lafage R., Schwab F.J., Lafage V. (2015). Sagittal Alignment of the Spine: What Do You Need to Know?. Clin. Neurol. Neurosurg..

[12] Zhang H., Zhang Z., Wang Z., Cheng J., Wu Y., Fan Y., Wang T., Wang Z. (2017). Optimal Pelvic Incidence Minus Lumbar Lordosis Mismatch after Long Posterior Instrumentation and Fusion for Adult Degenerative Scoliosis. Orthop. Surg..

[13] Pecina M., Dapic T. (2007). More than 20-Year Follow-up Harrington Instrumentation in the Treatment of Severe Idiopathic Scoliosis. Eur. Spine J..

[14] Glassman S.D., Berven S., Bridwell K., Horton W., Dimar J.R. (2005). Correlation of Radiographic Parameters and Clinical Symptoms in Adult Scoliosis. Spine.

[15] Barrey C., Jund J., Noseda O., Roussouly P. (2007). Sagittal Balance of the Pelvis-Spine Complex and Lumbar Degenerative Diseases. A Comparative Study about 85 Cases. Eur. Spine J..

[16] Protopsaltis T., Schwab F., Bronsard N., Smith J.S., Klineberg E., Mundis G., Ryan D.J., Hostin R., Hart R., Burton D. (2014). TheT1 Pelvic Angle, a Novel Radiographic Measure of Global Sagittal Deformity, Accounts for Both Spinal Inclination and Pelvic Tilt and Correlates with Health-Related Quality of Life. J. Bone Jt. Surg. Am..

[17] Ryan D.J., Protopsaltis T.S., Ames C.P., Hostin R., Klineberg E., Mundis G.M., Obeid I., Kebaish K., Smith J.S., Boachie-Adjei O. (2014). T1 Pelvic Angle (TPA) Effectively Evaluates Sagittal Deformity and Assesses Radiographical Surgical Outcomes Longitudinally. Spine.

[18] Yang M., Yang C., Xu Z., Chen Z., Wei X., Zhao J., Shao J., Zhang G., Zhao Y., Ni H. (2016). Role of T1 Pelvic Angle in Assessing Sagittal Balance in Outpatients with Unspecific Low Back Pain. Medicine.

[19] Sheikh Alshabab B., Gupta M.C., Lafage R., Bess S., Shaffrey C., Kim H.J., Ames C.P., Burton D.C., Smith J.S., Eastlack R.K. (2021). Does Achieving Global Spinal Alignment Lead to Higher Patient Satisfaction and Lower Disability in Adult Spinal Deformity?. Spine.

[20] Ames C.P., Smith J.S., Pellisé F., Kelly M., Alanay A., Acaroğlu E., Pérez-Grueso F.J.S., Kleinstück F., Obeid I., Vila-Casademunt A. (2019). Artificial Intelligence Based Hierarchical Clustering of Patient Types and Intervention Categories in Adult Spinal Deformity Surgery: Towards a New Classification Scheme That Predicts Quality and Value. Spine.

